# Second Law Analysis of Dissipative Flow over a Riga Plate with Non-Linear Rosseland Thermal Radiation and Variable Transport Properties

**DOI:** 10.3390/e20080615

**Published:** 2018-08-18

**Authors:** Muhammad Idrees Afridi, Muhammad Qasim, Abid Hussanan

**Affiliations:** 1 Department of Mathematics, COMSATS Institute of Information Technology, Park Road, Chak Shahzad, Islamabad 44000, Pakistan; 2 Division of Computational Mathematics and Engineering, Institute for Computational Science, Ton Duc Thang University, Ho Chi Minh City 700000, Vietnam; 3 Faculty of Mathematics and Statistics, Ton Duc Thang University, Ho Chi Minh City 700000, Vietnam

**Keywords:** entropy generation, heat transfer, variable transport properties, Riga plate, viscous and magnetic dissipation, non-linear Rosseland thermal radiations

## Abstract

In this article, we investigated entropy generation and heat transfer analysis in a viscous flow induced by a horizontally moving Riga plate in the presence of strong suction. The viscosity and thermal conductivity of the fluid are taken to be temperature dependent. The frictional heating function and non-linear radiation terms are also incorporated in the entropy generation and energy equation. The partial differential equations which model the flow are converted into dimensionless form by using proper transformations. Further, the dimensionless equations are reduced by imposing the conditions of strong suction. Numerical solutions are obtained using MATLAB boundary value solver bvp4c and used to evaluate the entropy generation number. The influences of physical flow parameters arise in the mathematical modeling are demonstrated through various graphs. The analysis reveals that velocity decays whereas entropy generation increases with rising values of variable viscosity parameter. Furthermore, entropy generation decays with increasing variable thermal conductivity parameter.

## 1. Introduction

In the classical MHD flow control, the boundary layer flow of an electrically conducting fluid can be controlled by the application of an external magnetic field subjected to the condition that the electric conductivity of fluid should be high (e.g., liquid form of semiconductors, plasma, electrolytes and liquid metals). Due to the high electric conductivity of the fluid, the influence of applied external magnetic field is significant even in presence of moderate strength of the magnetic field (~1 Tesla). In addition, the application of an external electric field is not required in order to achieve an efficient flow control. In case of weakly conducting fluids (e.g., sea water) the electric current induced by the external magnetic field is too small and external electric field must be applied to control the flow separation. The Lorentz force parallel to the wall has the ability to stabilize the motion inside the boundary layer by slowing down its growth. Gailitis and Lielausis [1] proposed for the first time an ingenious way to produce the wall-parallel Lorentz force. The flow control device developed by Gailitis and Lielausis [1] consists of alternative permanent magnetics and electrodes of equal width. Later on, Avilov [2] called it the Riga plate [2]. Perhaps, for the very first time, Tsinober and Shtern [3] used the Grinberg-term in the momentum equation to analyze the boundary layer flow. The Grinberg-term is free from flow velocity and decays exponentially with the distance normal to the main flow. The influence of suction/injection on classical Blasius and Sakiadis flow over a Riga plate is investigated by Pantokratoras [4]. Magyari and Pantokratoras [5] reported the mixed convection flow of Newtonian fluid induced by the Riga plate. Ahmed et al. [6] employed the perturbation technique and numerical simulation to study the mixed convection flow of nanofluid past over a Riga plate. The effects of non-linear thermal radiation on the Blasius and Sakiadis flow of nanofluids over a Riga plate by taking the effects of Brownian diffusion and thermophoresis is studied by Ramesh and Gireesha [7]. Pantokratoras and Magyari [8] numerically investigated the free convection flow over a Riga plate by using the tridiagonal matrix algorithm.

The non-linear thermal radiation has major importance in the high-temperature processes. The linear thermal radiation approximation is valid in the low-temperature processes. The effects of non-linear Rosseland thermal radiation on the classical Sakiadis and Blasius flows are investigated by Pantokratoras and Fang [9,10]. Recently, Afridi and Qasim [11] reported the influences of non-linear thermal radiation on heat transfer and entropy production in a fluid flow over a horizontally moving thin needle. Sithole et al. [12] examined the viscous dissipation and non-linear radiation impacts on the entropy generation rate in a second grade nanofluid flow over an elastic stretching sheet. The effects of variable viscosity and nonlinear thermal radiation on bio-convection flow by taking gyrotactic microorganisms in the presence of Lorentz force are reported by Babu and Sandeep [13]. Very recently Ghadikolaei et al. [14] studied the Casson fluid flow over a permeable inclined stretching surface by incorporated the influence of magnetic field.

In the case of high-temperature processes, it is more convincing to consider the viscosity and thermal conductivity to be temperature dependent. The influence of temperature dependent viscosity on mixed convection flow is reported by Hossain and Munir [15]. Khader and Megahed [16] performed the first law analysis of viscous fluid flow over a slendring stretching surface by taking the temperature-dependent thermal conductivity and linear thermal radiation. Mureithi et al. [17] found that the variable viscosity parameter has substantial impacts on temperature and velocity distribution inside the boundary layer. The combined effects of variable thermal conductivity and variable viscosity on a mixed convection flow under the impact of Lorentz force is investigated by Pal and Mondal [18]. Their investigation reveals that temperature profile enhances with enhancing values of variable thermal conductivity parameter. Manjunatha and Gireesha [19] studied dusty fluid flow with variable viscosity and thermal conductivity under the influence of magnetic force.

From an industrial point of view, the analysis of heat transfer in boundary layer flows is of great importance [20,21,22]. In the recent past, the heat transfer analyses in industrial processes involving either closed or open system are confined to first law analysis. The main purpose of the first law analysis is to find the temperature distribution inside the thermodynamic system and the rate of heat flux at the solid boundary [23,24,25]. This is a well-known fact that in all real thermodynamic processes the quantity of energy is conserved but the quality of energy reduces [26]. The reduction in quality of energy in thermodynamic processes is measured by entropy generation. In other words, the quality of energy decays with the enhancement of entropy generation in a process. There are many causes of entropy generation such as fluid friction, mixing, dissipative forces, heat transfer and unrestrained expansion etc. The aim of the second law analysis is to minimize the entropy generation in a thermodynamic system. Bejan [27] pulled out the way to reduce the entropy generation in a convective heat transfer problem and called it entropy generation minimization (EGM). After the innovative work of Bejan [27], many researchers used the second law of thermodynamics to minimize the entropy generation. Some of the recent studies on the second law analysis are referenced in [28,29,30,31,32,33,34,35].

The aim of the present study is to investigate the flow and heat transfer analysis of the dissipative flow induced by a horizontally moving Riga plate in a quiescent fluid with variable transport properties and non-linear Rosseland thermal radiations. The second law analysis is also performed in the presence of viscous dissipation. The governing equations are non-dimentionalized with the help of suitable transformations. The dimensionless equations are further simplified by using the assumption of strong suction. The reduced set of governing equations is solved numerically by utilizing MATLAB built-in boundary value solver bvp4c. The variations of quantities of interests with emerging dimensionless numbers are portrayed graphically and discussed physically in detail. To the best of author’s knowledge, such an analysis is not reported before.

## 2. The Mathematical Model

**Figure 1:**
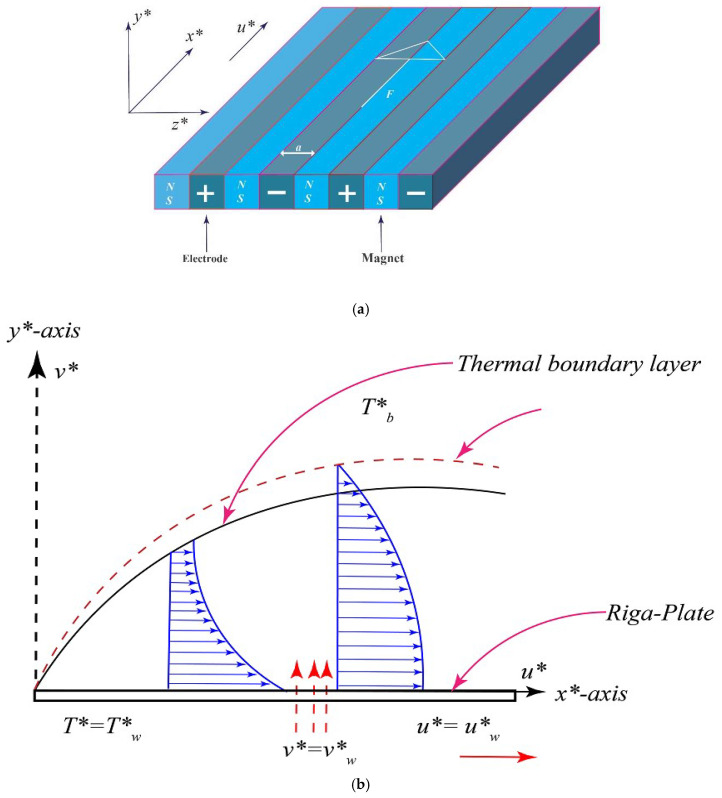
(**a**) Sketch of Riga plate with coordinates system; (**b**) Sketch of the flow showing the velocity and temperature profile.

An incompressible boundary layer flow over a Riga plate moving horizontally in a quiescent electrically conducting fluid is considered. The temperature of the ambient fluid and the velocity of Riga plate are assumed to be constant and denoted by $T_{b}^{*}$ and $u_{w}^{*}$, respectively. The thermal conductivity and viscosity of the fluid are assumed to be temperature dependent. Figure 1a,b respectively show the Riga plate (also known as an electromagnetic actuator) which consists of permanent magnets and electrodes of equal width $a_{o}$ and sketch of the velocity and temperature profile. In addition, the temperature of the surface of Riga plate $T_{w}^{*}$ is supposed to be constant such that $T_{w}^{*}>T_{b}^{*}$ (heated Riga Plate). Based upon the above flow assumptions, the governing equations in the presence of non-linear thermal radiation and viscous dissipation take the following form:

(1) $$\frac{\partial u^{*}}{\partial x^{*}}+\frac{\partial v^{*}}{\partial y^{*}}=0,\;\;$$

(2) $$\rho \left(u^{*}\frac{\partial u^{*}}{\partial x^{*}}+v^{*}\frac{\partial u^{*}}{\partial y^{*}}\right)=\frac{\partial \mu \left(T^{*}\right)}{\partial T^{*}}\frac{\partial T^{*}}{\partial y^{*}}\frac{\partial u^{*}}{\partial y^{*}}+\mu \left(T^{*}\right)\;\frac{\partial^{2}u^{*}}{\partial y^{*2}}+\frac{\pi j_{o}M^{*}}{8}e^{\left(-\frac{\pi y^{*}}{a_{o}}\right)},\;$$

(3) $$\begin{array}{ll} u^{*}\frac{\partial T^{*}}{\partial x^{*}}+v^{*}\frac{\partial T^{*}}{\partial y^{*}}= & \frac{1}{\rho c_{p}}\left[\frac{\partial k\left(T^{*}\right)}{\partial y^{*}}{\left(\frac{\partial T^{*}}{\partial y^{*}}\right)}^{2}+k\left(T^{*}\right)\;\frac{\partial^{2}T^{*}}{\partial y^{*2}}\right]+\frac{16\sigma_{SB}}{3a_{R}\rho c_{p}}\frac{\partial }{\partial y^{*}}\left(T^{*3}\frac{\partial T^{*}}{\partial y^{*}}\right)\;\\ & +\frac{\mu \left(T^{*}\right)}{\rho c_{p}}{\left(\frac{\partial u^{*}}{\partial y^{*}}\right)}^{2}, \end{array}\;$$

subject to the boundary conditions:

(4) $$u^{*}\left(x^{*},\;0\right)=u_{w}^{*},\;v^{*}\left(x^{*},\;0\right)=v_{w}^{*},\;T^{*}\left(x^{*},\;0\right)=T_{w}^{*},\;$$

(5) $$u^{*}\left(x^{*},\;y^{*}\to \infty \right)\to 0,\;T^{*}\left(x^{*},\;y^{*}\to \infty \right)\to T_{b}^{*}.\;$$

where $<u^{*},$ $v^{*}>$ represent velocity components in the direction of $x^{*}-axis$ and $y^{*}-axix.$ respectively, $T^{*}$ shows fluid temperature inside the boundary layer, $\mu \left(T^{*}\right)=\left(\frac{\mu_{b}}{1+\mu_{o}\left(T^{*}-T_{b}^{*}\right)}\right)$ and $k\left(T^{*}\right)=k_{b}\left(1+\varepsilon \frac{T^{*}-T_{b}^{*}}{T_{w}^{*}-T_{b}^{*}}\right)$ are temperature dependent viscosity and thermal conductivity of the fluid, respectively. ε is a variable thermal conductivity parameter, $a_{o}$ represents the width of magnets and electrodes, $M^{*}$ is the magnetization of the permanent magnets, $j_{o}$ indicates the applied current density in the electrodes, $\sigma_{SB}$ is the Stefan-Boltzmann constant and $a_{R}$ is the Rosseland mean absorption coefficient.

Introducing the non-dimensional quantities:

(6) $$\frac{x^{*}}{l}=x,\;\frac{y^{*}}{L}=y,\;\frac{u^{*}}{u_{w}^{*}}=u,\;\frac{v^{*}}{v_{o}}=v,\;\theta =\frac{T^{*}-T_{b}^{*}}{T_{w}^{*}-T_{b}^{*}},\;\;$$

(7) $$l=\frac{u_{w}^{*}L^{2}}{\vartheta_{b}},\;L=\frac{a_{o}}{\pi },\;v_{o}=\frac{\pi \vartheta_{b}}{a_{o}},\;\vartheta_{b}=\frac{\mu_{b}}{\rho },\;\mathrm{Pr}=\frac{\vartheta_{b}\rho c_{p}}{k_{b}},\;M=\frac{a_{o}^{2}j_{o}M^{*}}{8\pi u_{w}^{*}\rho \vartheta_{b}}\;$$

(8) $$Ec=\frac{u_{w}^{*2}}{c_{p}\left(T_{w}^{*}-T_{b}^{*}\right)}\;(\mathrm{Eckert}\;\mathrm{number}),\;N_{r}=\frac{a_{R}k_{b}}{4\sigma_{SB}T_{b}^{*3}}\;(\mathrm{thermal}\;\mathrm{radiation}\;\mathrm{parameter})$$

into Equations (1)–(5), we have:

(9) $$\frac{\partial u}{\partial x}+\frac{\partial v}{\partial y}=0,\;\;$$

(10) $$u\frac{\partial u}{\partial x}+v\frac{\partial u}{\partial y}=\frac{1}{1+\delta \theta }\frac{\partial^{2}u}{\partial y^{2}}-\frac{\delta }{{\left(1+\delta \theta \right)}^{2}}\frac{\partial \theta }{\partial y}\frac{\partial u}{\partial y}+\frac{1}{1+\delta \theta }\frac{\partial^{2}u}{\partial y^{2}}+Me^{\left(-y\right)},\;\;$$

(11) $$\begin{array}{ll} u\frac{\partial \theta }{\partial x}+v\frac{\partial \theta }{\partial y} & =\frac{1}{\mathrm{Pr}}\left(\varepsilon {\left(\frac{\partial \theta }{\partial y}\right)}^{2}+\left(1+\varepsilon \theta \right)\;\frac{\partial^{2}\theta }{\partial y^{2}}\right)+\frac{1}{3\mathrm{Pr}N_{r}\left(\theta_{r}-1\right)}\frac{\partial^{2}}{\partial y^{2}}{\left(\theta \left(\theta_{r}-1\right)+1\right)}^{4}\\ & +\frac{Ec}{1+\delta \theta }{\left(\frac{\partial u}{\partial y}\right)}^{2}, \end{array}\;$$

(12) $$u=1,\;v=\frac{v_{w}^{*}}{v_{o}}=v_{w},\;\theta =1,\;\mathrm{at}\;y=0,\;\;$$

(13) u→0,  θ→0 as y→∞. 

By using the assumption of strong suction [6], Equations (9)–(11) can be rewritten in the following form:

(14) $$\frac{\partial v}{\partial y}=0,\;\;$$

(15) $$v\frac{\partial u}{\partial y}=\frac{1}{1+\delta \theta }\frac{\partial^{2}u}{\partial y^{2}}-\frac{\delta }{{\left(1+\delta \theta \right)}^{2}}\frac{\partial \theta }{\partial y}\frac{\partial u}{\partial y}+\frac{1}{1+\delta \theta }\frac{\partial^{2}u}{\partial y^{2}}+Me^{\left(-y\right)},\;$$

(16) $$\begin{array}{ll} v\frac{\partial \theta }{\partial y} & =\frac{1}{\mathrm{Pr}}\left(\varepsilon {\left(\frac{\partial \theta }{\partial y}\right)}^{2}+\left(1+\varepsilon \theta \right)\;\frac{\partial^{2}\theta }{\partial y^{2}}\right)+\frac{1}{3\mathrm{Pr}N_{r}\left(\theta_{r}-1\right)}\frac{\partial^{2}}{\partial y^{2}}{\left(\theta \left(\theta_{r}-1\right)+1\right)}^{4}\\ & +\frac{Ec}{1+\delta \theta }{\left(\frac{\partial u}{\partial y}\right)}^{2}. \end{array}\;$$

The condition $v=v_{w}$ at  y=0 and continuity equation gives $v=v_{w},$ thus Equations (15) and (16) can be written as:

(17) $$v_{w}\frac{\partial u}{\partial y}=\frac{1}{1+\delta \theta }\frac{\partial^{2}u}{\partial y^{2}}-\frac{\delta }{{\left(1+\delta \theta \right)}^{2}}\frac{\partial \theta }{\partial y}\frac{\partial u}{\partial y}+\frac{1}{1+\delta \theta }\frac{\partial^{2}u}{\partial y^{2}}+Me^{\left(-y\right)},\;\;$$

(18) $$\begin{array}{ll} v_{w}\frac{\partial \theta }{\partial y} & =\frac{1}{\mathrm{Pr}}\left(\varepsilon {\left(\frac{\partial \theta }{\partial y}\right)}^{2}+\left(1+\varepsilon \theta \right)\;\frac{\partial^{2}\theta }{\partial y^{2}}\right)+\frac{1}{3\mathrm{Pr}N_{r}\left(\theta_{r}-1\right)}\frac{\partial^{2}}{\partial y^{2}}{\left(\theta \left(\theta_{r}-1\right)+1\right)}^{4}\\ & +\frac{Ec}{1+\delta \theta }{\left(\frac{\partial u}{\partial y}\right)}^{2}.\; \end{array}\;$$

## 3. Entropy Generation

By assuming a viscous incompressible fluid element of a finite size such that it acts like an open thermodynamic system and by employing the second law of thermodynamics, the volumetric rate of entropy generation $\left({{\dot{E}}^{\prime \prime \prime }}_{Gen}\right)$ in the presence of non-linear Rosseland thermal radiation and viscous dissipation takes the following form:

(19) $${\dot{E}}_{Gen}^{\prime \prime \prime }=\frac{k\left(T^{*}\right)}{T^{*2}}{\left(\frac{\partial T^{*}}{\partial y^{*}}\right)}^{2}+\frac{\mu \left(T^{*}\right)}{T^{*}}{\left(\frac{\partial u^{*}}{\partial y^{*}}\right)}^{2}+k\left(T^{*}\right)\frac{16\sigma_{SB}T^{*}}{3a_{R}k\left(T^{*}\right)}{\left(\frac{\partial T^{*}}{\partial y^{*}}\right)}^{2}\;$$

Using the transformations defined in Equations (6)–(8) we obtained the dimensionless form of volumetric rate of entropy generation Ns as given below

(20) $$\begin{array}{l} Ns=\frac{{\dot{E}}_{Gen}^{\prime \prime \prime }}{{\left({\dot{E}}_{Gen}^{\prime \prime \prime }\right)}_{o}}\\ \overset{}{=\underset{Thermal\;irreversibility}{\underset{︸}{{\left(\theta_{r}-1\right)}^{2}\left[\frac{1+\varepsilon \theta }{{\left(\theta \left(\theta_{r}-1\right)+1\right)}^{2}}+\frac{4}{3N_{r}}\left(\theta \left(\theta_{r}-1\right)+1\right)\right]\;{\left(\frac{\partial \theta }{\partial y}\right)}^{2}}}}\\ +\underset{Frictional\;irreversibility}{\underset{︸}{\frac{Ec\mathrm{Pr}\left(\theta_{r}-1\right)}{\left(1+\delta \theta \right)\left(\theta \left(\theta_{r}-1\right)+1\right)}{\left(\frac{\partial u}{\partial y}\right)}^{2}}} \end{array}\;$$

here ${\left({\dot{E}}_{Gen}^{\prime \prime \prime }\right)}_{o}=\frac{k_{b}}{L^{2}}$ is the characteristic entropy generation, $\theta_{r}=\frac{T_{w}^{*}}{T_{b}^{*}}$ denotes the heating parameter, $\delta =\mu_{o}\left(T_{w}^{*}-T_{b}^{*}\right)$ indicates a parameter related to variable viscosity.

## 4. Results and Discussion

**Figure 2:**
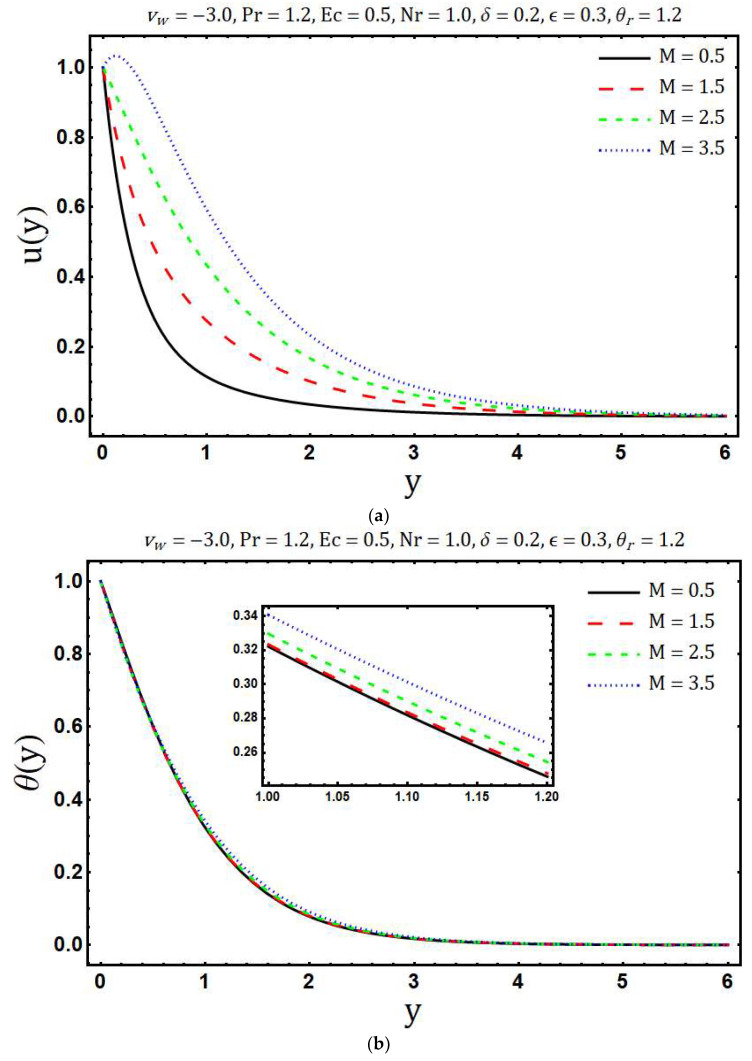

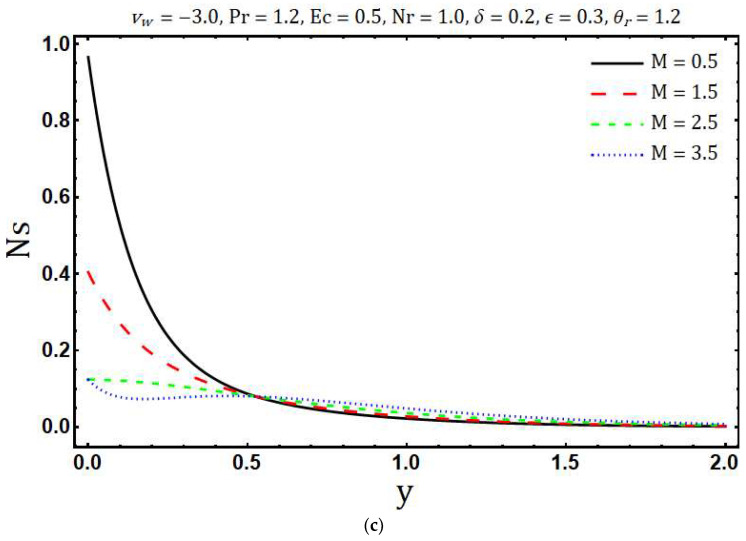
Effects of M on (**a**) velocity profile (**b**) temperature distribution and (**c**) entropy generation.

**Figure 3:**
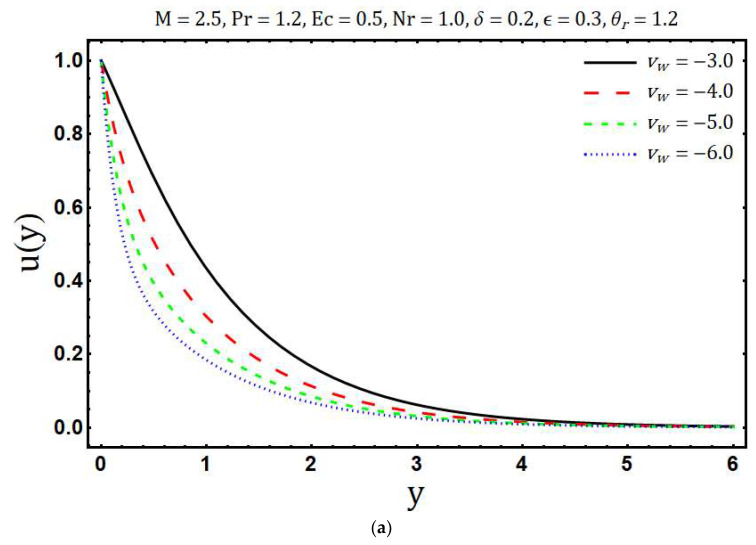

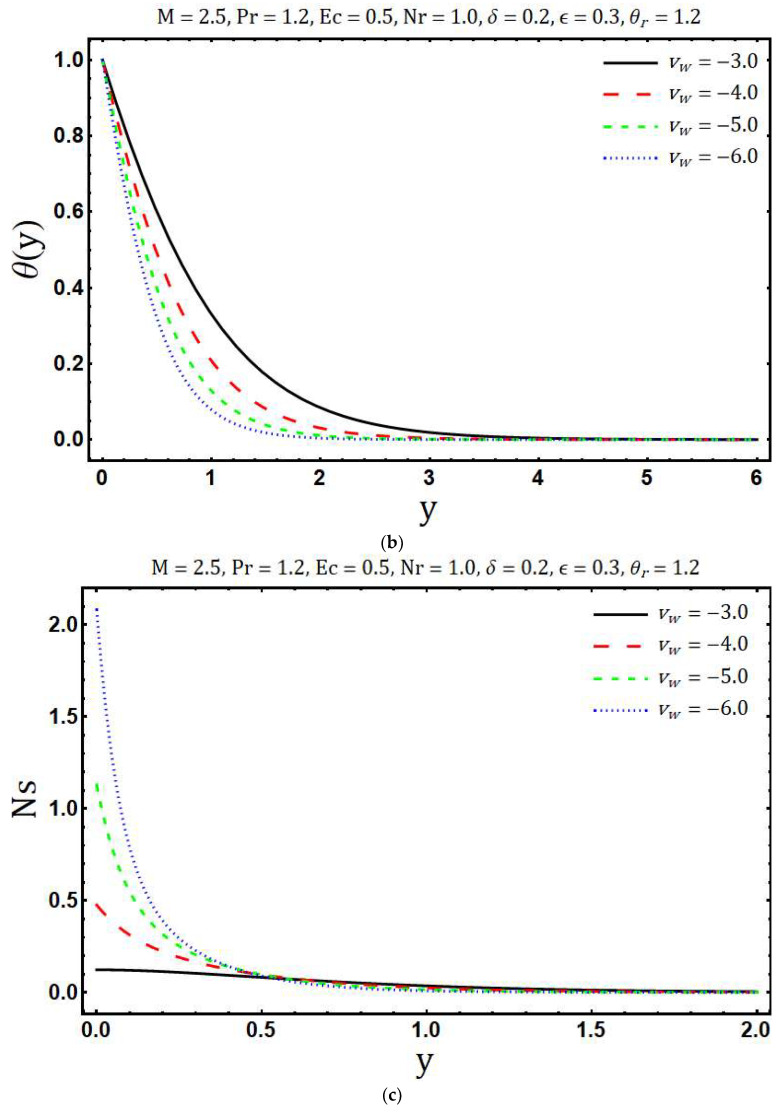
Effects of $v_{w}$ on (**a**) velocity profile (**b**) temperature distribution and (**c**) entropy generation.

**Figure 4:**
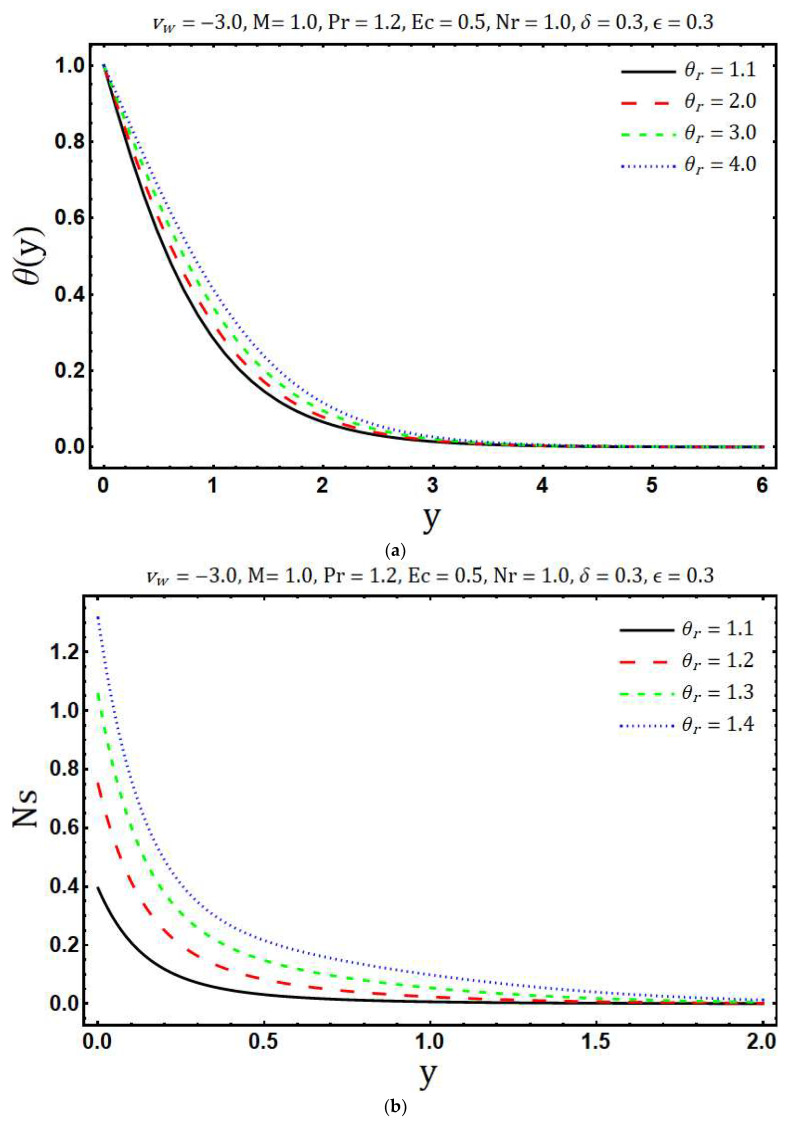
Effects of $\theta_{r}$ on (**a**) temperature distribution and (**b**) entropy generation.

**Figure 5:**
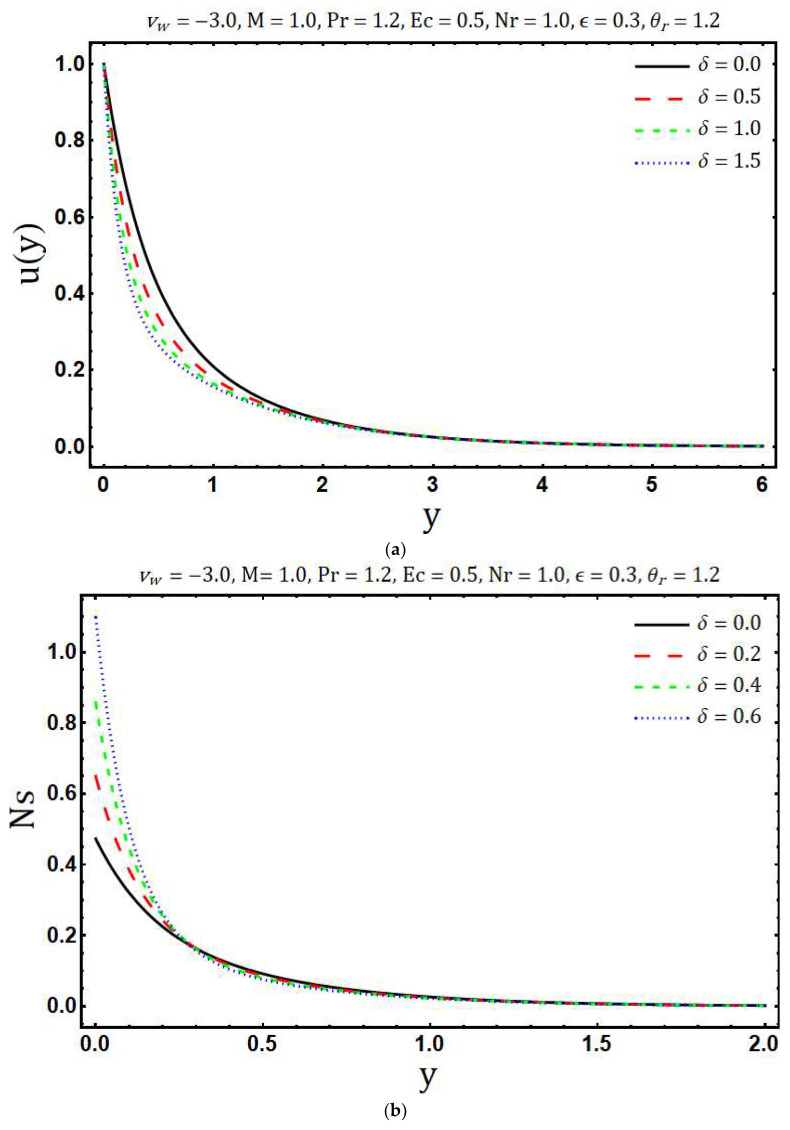
Effects of δ on (**a**) velocity profile and (**b**) entropy generation.

**Figure 6:**
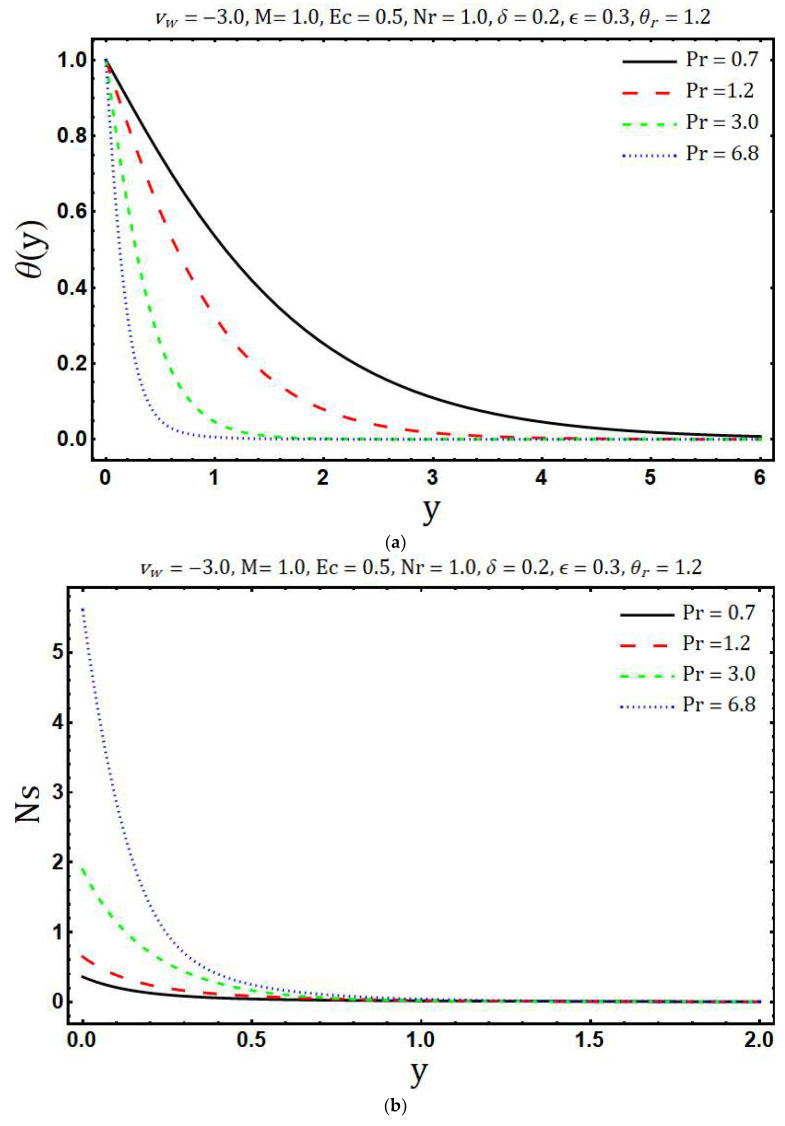
Effects of Pr on (**a**) temperature distribution and (**b**) entropy generation.

The numerical solutions of the system of Equations (17) and (18) with the corresponding boundary conditions (12, 13) are obtained using Matlab in-built boundary value solver bvp4c. The numerical values of ${\left(\frac{\partial \theta }{\partial y}\right)}_{y=0}$ are tabulated in Table 1. This table shows that the numerical values obtained by using bvp4c and shooting method are sufficiently close to each other, which validates our current numerical procedure. The obtained numerical solutions are used to examine the behavior of entropy generation against the various embedding physical parameters. Figure 2a shows the variations in velocity profile for different values of the modified Hartmann number *M*. Here we observed that velocity of the fluid *u*(*y*) accelerates with an increase in modified Hartmann number. This is consistent with the physics of the problem because *M* > 0 implies adding flow mechanism on the velocity profile. Figure 2b reflects the variation of temperature profile with increasing modified Hartmann number. It is revealed that an increase occurs in the temperature profile with rising values of modified Hartmann number. The variations in entropy generation corresponding to different values of the modified Hartmann number is shown in Figure 2c. A reduction in entropy generation is noticed with the enhancement of a modified Hartmann number. Whereas this behavior is reversed after a certain vertical distance from a Riga plate. Figure 3a elaborates the effects of the mass suction parameter *ν_w_* on *u*(*y*). The decrease in fluid velocity is observed with increasing mass suction parameter. Physically, suction pulls the fluid towards the surface of the Riga plate and this pulling acts as a retarding force, consequently, velocity decays. Further, the thickness of the viscous boundary layer decays with the increasing mass suction parameter *ν_w_*. The ability of mass suction parameter *ν_w_* to reduce the thermal boundary layer is clearly seen from Figure 3b. In addition, the temperature also decays and asymptotically approaches to zero towards the edge of the temperature boundary layer. The enhancement in the entropy generation with increasing mass suction parameter *ν_w_* is shown in Figure 3c. Physically, this is because of increasing thermal and velocity gradients with increasing mass suction parameter. Figure 4a demonstrates the variations of temperature *θ*(*y*) against the rising values of the heating parameter *θ_r_*. It is noticed that temperature *θ*(*y*) rises with the increasing values of *θ_r_*. The increasing behavior of temperature is expected because *θ_r_* increases with increasing the operating temperature difference $T_{w}^{*}-T_{b}^{*}$ and consequently the fluid temperature rises. It is noticed from Figure 4b that the increase in heating parameter enhances the entropy generation. The heating of fluid due to increased heating parameter causes more entropy generation. Further, the effects are significant at the surface of Riga plate and its proximity. Figure 5a presented the influence of variable viscosity parameter *δ* on the velocity profile *u*(*y*). It is found that *u*(*y*) decays with variable viscosity parameter *δ*. The influence of *δ* on entropy generation number Ns is depicted in Figure 5b. The entropy generation increase with the rising values of *δ*. This is due to the increasing velocity gradients with the increasing values of *δ*. The enhancement in entropy generation is prominent at the surface and near the surface of the Riga plate. Additionally, maximum entropy generation is observed at the surface of the Riga plate. The increasing trend of Ns is reversed after a certain distance y. Figure 6a presents the variations in temperature *θ*(*y*) corresponding to increasing values of Prandtl number Pr. The influence of Pr is to decrease the fluid temperature. Physically, with growing values of Pr the thermal diffusivity reduces which is responsible for the decay of temperature profile. Figure 6b presents the distribution of entropy generation for against the multiple values of the Prandtl number. It is clearly seen that Ns enhances if Pr increases. Physically, the thermal gradients increase with growing values of Pr and so the entropy generation increases.

**Figure 7:**
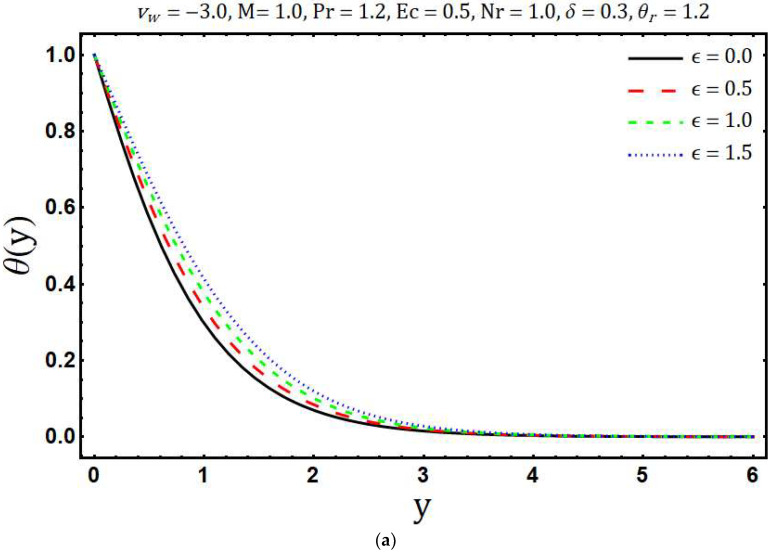

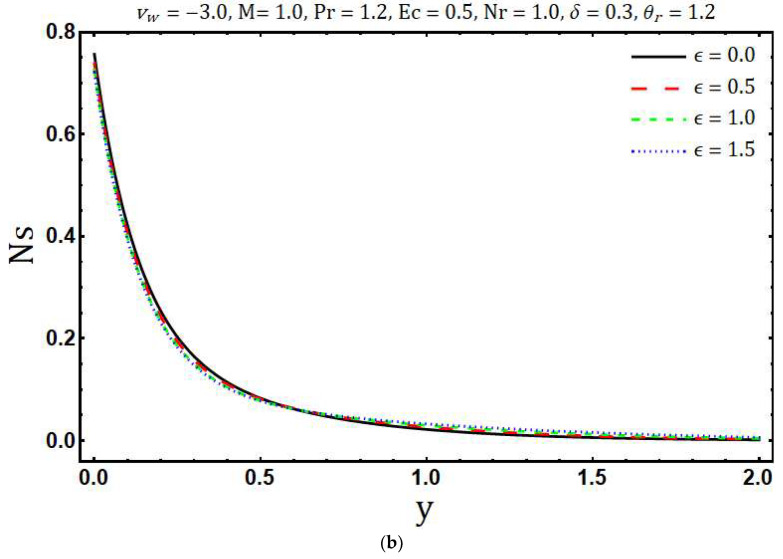
Effects of ε on (**a**) temperature distribution and (**b**) entropy generation.

**Figure 8:**
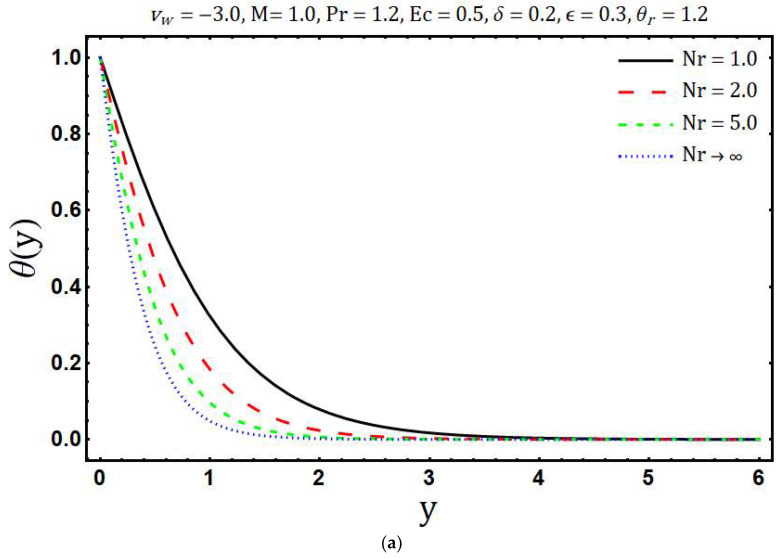

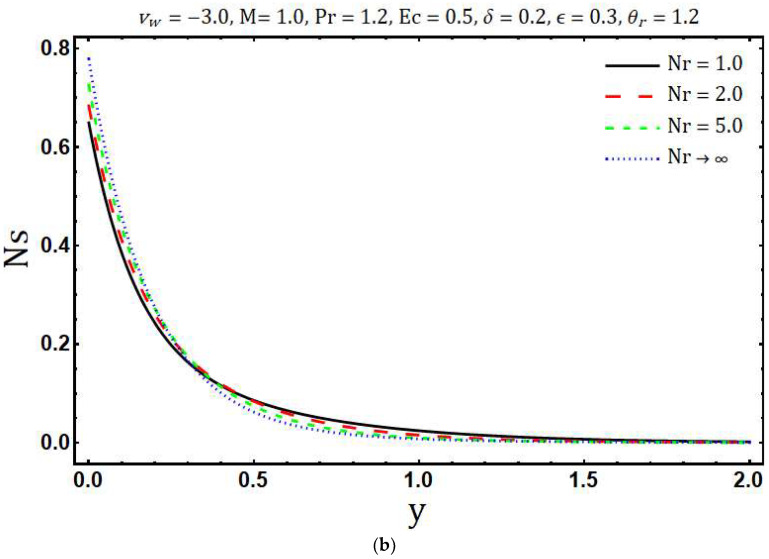
Effects of $N_{r}$ on (**a**) temperature distribution and (**b**) entropy generation.

**Figure 9:**
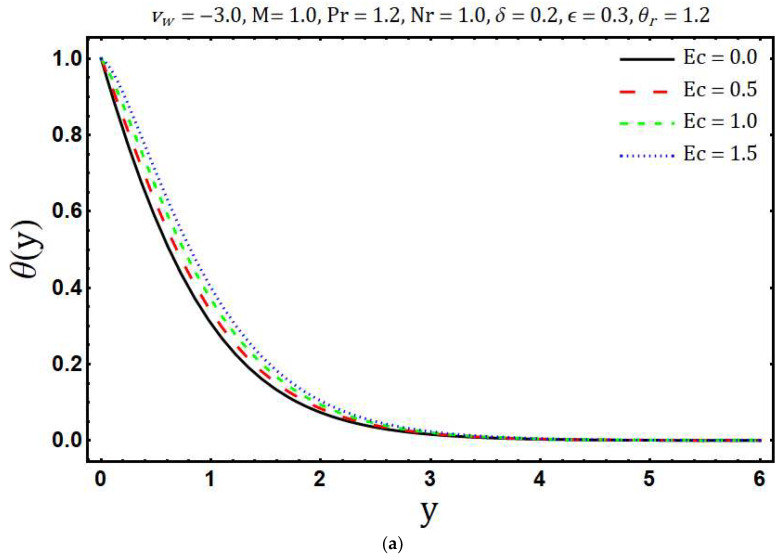

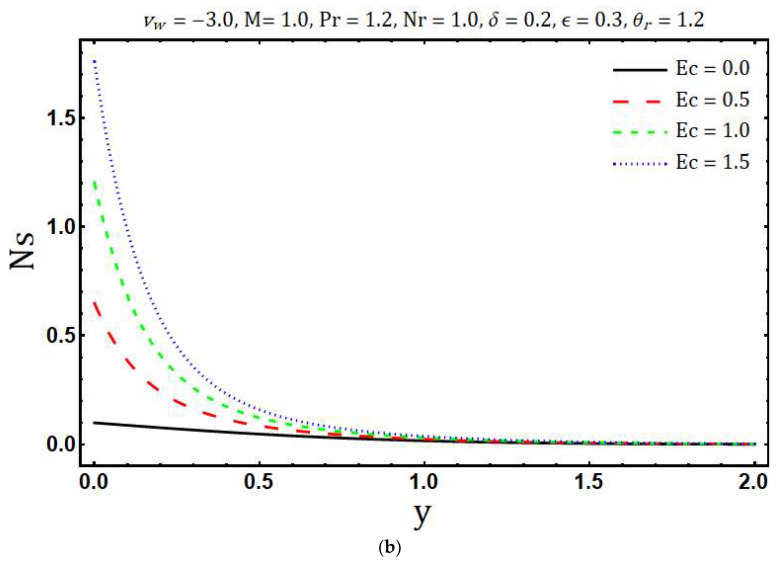
Effects of Ec on (**a**) temperature distribution and (**b**) entropy generation.

Figure 7a portrays the effects of variable thermal conductivity parameter ε on temperature θ(y). It is found that the temperature θ(y) and thickness of the thermal boundary layer increase if ε increases. This is due to the fact that thermal conductivity increases with growing values of ε which in turn enhances the thermal energy penetration. The influence of ε on the entropy generation is shown in Figure 7b. The ε tends to decrease the entropy generation and this is because of the decreasing thermal gradients with rising values of ε. Impacts of thermal radiation parameter $N_{r}$ on temperature θ(y) are presented in Figure 8a. It is found that as the values of $N_{r}$ increases, θ(y) decreases. Physically, for a given value of $k_{b}$ and $a_{R}$, an increase in $N_{r}=\frac{k_{b}a_{R}}{4\sigma_{SB}T_{b}^{*3}}$ yields a decrease in the ambient temperature $T_{b}^{*}$ and this means that significant part of the fluid inside the boundary layer has low temperature and consequently the thermal diffusivity $\left(\alpha +\frac{16\sigma_{SB}}{3\rho c_{p}a_{R}}T^{3}\right)$ becomes low with the thin thermal boundary layer. The effect of $N_{r}$ on distribution of entropy generation in the main flow region is depicted in Figure 8b. It is clearly seen that Ns enhances with growing values of $N_{r}$ whereas opposite trend is observed after a certain transverse distance y. Further, the surface of the Riga plate is the strong source of entropy generation due to the large thermal and velocity gradients. In Figure 9a temperature θ(y) is plotted against the transversal distance y for different values of the Eckert number Ec. The increase in θ(y) is observed with the increasing values of Ec and this is because of dissipative frictional forces between the fluid layer. The influence of different increasing values of Ec on Ns is illustrated in Figure 9b. It is found that an increase in the Ec leads to enhance the Ns. Physically, the dissipative frictional forces between the fluid layer increase with increasing Eckert number and consequently entropy generation enhances.

## 5. Concluding Remarks

Numerical computation has been done to examine the heat transfer and entropy generation in boundary layer flow over a Riga plate by considering the effects of non-linear thermal radiation with variable transport properties. The following are the main outcomes drawn from this study:

- Decay in the magnitude of velocity u(y) is found as the mass suction parameter $v_{w}$ and variable viscosity parameter increases δ while an enhancement in modified Hartmann number M accelerates the fluid motion.
- Temperature θ(y) increases with rising values of the Eckert number, heating parameter, and variable thermal conductivity while an opposite behavior has been observed for growing values of the mass suction parameter, Prandtl number, and radiation parameter $N_{r}$.
- The decrement in entropy generation Ns is observed with increasing values of modified Hartmann number and variable thermal conductivity while increment in Ns is observed with rising values of Prandtl number, radiation parameter, mass suction parameter, Eckert number, variable viscosity parameter and heating parameter.
- Maximum entropy is generated at the surface of Riga plate.

## Figures and Tables

**Table 1:** Comparison of the numerical values of ${\left(\frac{\partial \theta }{\partial y}\right)}_{y=0}$ for different embedding physical flow parameters.

								${\left(\frac{\partial \theta }{\partial y}\right)}_{y=0}\;$	
$v_{w}\;$	M	δ	ε	Pr	Ec	$N_{r}\;$	$\theta_{r}\;$	Shooting	bvp4c
−3.0	2.5	0.2	0.3	1.2	0.5	1.0	1.2	−0.93626	−0.93625
−4.0								−1.23309	−1.23310
−5.0								−1.51387	−1.51385
−3.0	1.0							−0.85674	−0.85672
	2.0							−0.92065	−0.92065
	3.0							−0.94096	−0.94097
	2.5	0.0						−0.93125	−0.93125
		0.5						−0.94241	−0.94242
		1.0						−0.95012	−0.95010
		0.3	0.0					−1.02348	−1.02350
			0.5					−0.88924	−0.88925
			1.0					−0.78617	−0.78616
			0.3	0.7				−0.54823	−0.54825
				1.2				−0.93847	−0.93847
				3.0				−2.33939	−2.33938
				1.2	0.0			−0.99889	−0.99890
					0.3			−0.96259	−0.96257
					0.6			−0.92643	−0.92645
					0.5	1.0		−0.93847	−0.93845
						2.0		−1.37779	−1.37780
						5.0		−1.91672	−1.91671
						1.0	1.1	−1.09966	−1.09967
							1.2	−0.93847	−0.93846
							1.3	−0.79999	−0.79999

## Nomenclature

$a_{o}$	with of electrodes and magnets (m)
$c_{p}$	specific heat at constant pressure (J/kg K)
Ec	Eckert number
${\dot{E}}_{Gen}^{\prime \prime \prime }$	volumetric rate of entropy generation (W/K)
${\left({\dot{E}}_{Gen}^{\prime \prime \prime }\right)}_{o}$	characteristic volumetric rate of entropy generation (W/K)
$j_{o}$	current density $\left({A/m}^{2}\right)$
$k\left(T^{*}\right)$	temperature dependent thermal conductivity (W/mK)
$k_{b}$	thermal conductivity of fluid outside the boundary layer (W/mK)
L	length unit (m)
$M^{*}$	magnetization of the magnets (Tesla)
M	modified Hartmann number
$N_{r}$	thermal radiation parameter
$N_{s}$	entropy generation number
Pr	Prandtl number
$T^{*}$	fluid temperature (K)
$T_{w}^{*}$	temperature at the surface of Riga-plate (K)
$T_{b}^{*}$	ambient temperature (K)
$u^{*}$	horizontal velocity $\left({\mathrm{ms}}^{-1}\right)$
u	dimensionless horizontal velocity
$v^{*}$	vertical component of velocity $\left({\mathrm{ms}}^{-1}\right)$
v	dimensionless vertical velocity
$x^{*}$	horizontal coordinate (m)
x	dimensionless horizontal coordinate
$y^{*}$	vertical coordinate (m)
y	dimensionless vertical coordinate
**Greek Symbols**	
ε	variable thermal conductivity parameter
δ	variable viscosity parameter
$\mu \left(T^{*}\right)$	dynamic viscosity (kg/ms)
$\mu_{b}$	dynamic viscosity of ambient fluid (kg/ms)
$\vartheta_{b}$	kinematic viscosity of ambient fluid $\left(m^{2}/s\right)$
ρ	fluid density $\left({\mathrm{kg}/m}^{3}\right)$
θ	dimensionless temperature
$\theta_{r}$	heating parameter
**Subscripts**	
w	condition on boundary
b	condition outside the boundary layer
